# Role of nuclear RNA in regulating chromatin structure and transcription

**DOI:** 10.1016/j.ceb.2019.03.007

**Published:** 2019-06

**Authors:** Davide Michieletto, Nick Gilbert

**Affiliations:** 1 MRC Human Genetics Unit, Institute of Genetics and Molecular Medicine, University of Edinburgh, Edinburgh EH4 2XU, UK; 2 School of Physics and Astronomy, University of Edinburgh, EH9 3FD, UK

## Abstract

The importance of three-dimensional chromatin organisation in genome regulation has never been clearer. But in spite of the enormous technological advances to probe chromatin organisation in vivo, there is still a lack of mechanistic understanding of how such an arrangement is achieved. Here we review emerging evidence pointing to an intriguing role of nuclear RNA in shaping large-scale chromatin structure and regulating genome function. We suggest this role may be achieved through the formation of a dynamic nuclear mesh that can exploit ATP-driven processes and phase separation of RNA-binding proteins to tune its assembly and material properties.

**Current Opinion in Cell Biology** 2019, **58**:120–125

This review comes from a themed issue on **Cell Nucleus**

Edited by **Naoko Imamoto** and **Daniel Larson**

For a complete overview see the Issue and the Editorial

Available online 19th April 2019

**https://doi.org/10.1016/j.ceb.2019.03.007**

0959-440X/© 2019 The Authors. Published by Elsevier Ltd. This is an open access article under the CC BY license (http://creativecommons.org/licenses/by/4.0/).

There has been a dramatic increase in the number of studies directed at understanding and quantifying large-scale chromatin organisation in the cell nucleus. Many of these adopted methods based on FISH or chromosome conformation capture (3C) [1], nowadays virtually indispensable to obtain high-resolution data on genome architecture. Yet, these tools are oblivious to the mechanistic causes dictating specific chromatin conformations. Thus, alongside the development of 3C-based methods, there is still an urgent need to develop experiments and models to shed mechanistic insight into the key molecular players that shape chromatin structure. One promising element in this picture is RNA: while its textbook role is that of facilitating the translation of the genetic code into proteins, there is a surprising lack of understanding for the existence, and the functional role, of a large mass of RNA which is retained in the nucleus in interphase (hereafter “nuclear RNA”). One of the best known examples of this alternative form of RNA is that of Xist, which is known to play a crucial architectural role in silencing the inactive X [2]. This prominent example is also highly descriptive of the common approach towards RNA-mediated chromatin regulation, i.e. by specialised instances and at particular genomic loci. On the contrary, recent evidence suggest that the role of nuclear RNA is spread at genome-wide level and should be addressed as such. In this article, we review recent attempts to advance our understanding of genome-wide chromatin regulation and organisation by nuclear RNA and finally discuss emerging views relating nuclear RNA to chromatin decompaction and transcription micro-environments.

## From static nuclear matrix to dynamic nuclear mesh

**Figure 1:**
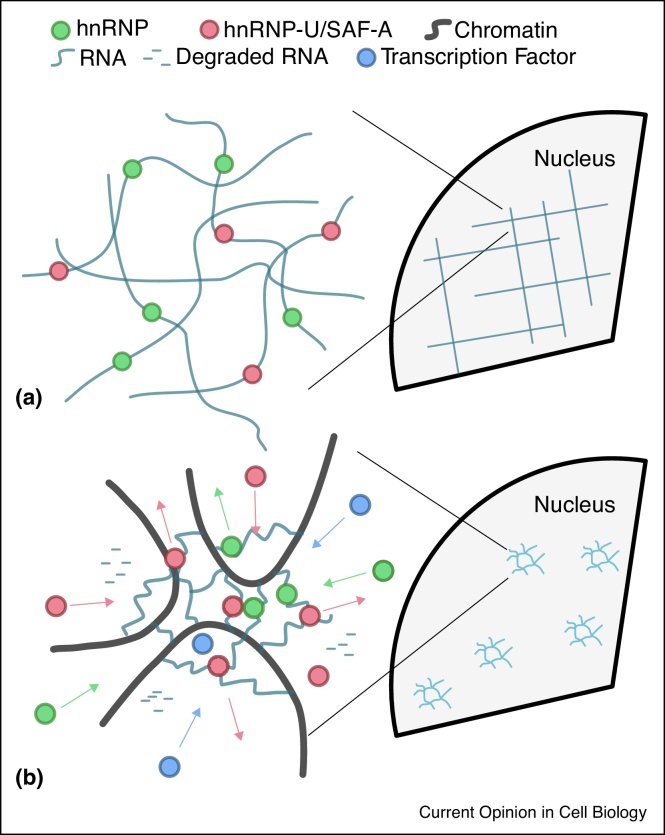
**(a)** Sketch of the original concept of static nuclear matrix made of hnRNP proteins and RNA. **(b)** New model based on nuclear RNA and SAF-A forming a dynamic, localised and recyclable scaffold which organises large-scale chromatin folding.

Nuclear extraction experiments in the 20th century suggested the existence of an extensive “nuclear matrix” which would permeate the cell nucleus even in the absence of chromatin [3] (Figure 1a). These experiments were performed in extreme conditions and no definitive proof in support of such static nuclear-spanning structure could be provided using more physiological approaches. Even though the idea of a static nuclear-spanning matrix contributing to chromatin organisation is now abandoned and largely surpassed, these experiments provided first evidence that nuclear proteins and RNA could play an architectural role in large-scale chromatin structure.

The family of heterogeneous nuclear ribonucleoproteins (hnRNP) was found to have a key role in forming the nuclear matrix and, in particular, scaffold attachment factor A (SAF-A, also called HNRNP-U) was identified as one sub-family of proteins with the highest affinity to scaffold attachment regions in the genome [4]. This protein is found across many cell types and its mutations have recently been associated with severe neurological disorders [5] and cancer [6]. HnRNPs are known to interact with nuclear RNA, in turn regulating the stabilisation and maturation of mRNA [7]; yet, their role as architectural elements of the genome is still poorly understood.

One recent development in this direction was the suggestion that nuclear RNAs can themselves act as regulatory factors and nuclear organisers [8]. For instance, long non-coding RNA (lncRNA) has been suggested to facilitate enhancer-promoter looping in cis, thus up-regulating the expression of nearby genes [9]. Other non-coding RNAs such as XIST, FIRRE and COT1 are abundant in the interphase nucleus. COT1 associates with euchromatin in interphase and is thought to maintain its decompacted state [10,11]; FIRRE and XIST co-localise with the inactive X-chromosome and determine its trans-chromosomal interactions [12,13]. Many lncRNAs are known to interact with hnRNP proteins, and in particular with SAF-A [12,10,14], yet the functional relevance of this interaction is not clear. A crucial element in this picture was added only recently by showing that SAF-A can regulate chromosome structure through interaction with nuclear RNA [15^••^].

A role for nuclear RNA in regulating chromatin structure is not well established, however much RNA is not exported to the cytoplasm (e.g. spliced out introns, other nuclear RNA species) and must presumably be degraded in the nucleus [16]. This implies that any nuclear structure that is assembled employing RNA cannot be static but constantly recycling degraded RNA with newly synthesised ones. In light of this, the original concept of a static nuclear matrix must be re-evaluated in terms of a dynamic scaffold possibly made of hnRNP and nuclear RNA that interfaces with the three-dimensional chromatin structure (Figure 1b).

## Global regulation by nuclear RNA

HnRNP proteins have been shown to associate non-coding and intronic RNAs [17,18]. For this reason, one may speculate that the self-assembly of a dynamic nuclear mesh would not be restricted to specific loci in the genome, but could be a general mechanism to regulate local chromatin architecture near actively transcribed loci. Regulation of transcription and chromosome structure by nuclear RNA is a long-standing topic [19,20] and both coding and non-coding RNAs have been postulated to possess a generic structural role in chromatin architecture for a long time [21,19,22]. Yet, a mechanical model linking transcription, nuclear RNA and hnRNP proteins is still missing. Here, we propose that hnRNP, and SAF-A in particular, guide the self-assembly of a dynamic RNA-based mesh (Figure 1). The precise mechanism of this self-assembly is still unclear but recent evidence indicates that SAF-A can switch between a monomeric and an oligomerised state upon ATP binding, and in the presence of RNA [15^••^]. In line with this finding, another study performing HiC and DamID in mouse hepatocytes after SAF-A depletion reports a global condensation of chromatin and compartment switching leading to an overall reduction in chromatin contacts [23^•^]. While the model of a self-assembled dynamic mesh is intriguing, it may be over-simplified. For instance, one element in this picture that is missing is the observation that hnRNP proteins often display intrinsically disordered regions (IDR, also known as low complexity domains [24]). As a consequence, they can undergo phase separation under a range of physiological conditions [25], i.e. they can convert from a mixed and uniform state into a demixed one whereby (spherical) condensates display higher internal density than their surroundings. The functional role of this phenomenon, whether relevant for regulating nuclear organisation, remains unclear and a topic of intense research [26].

## Phase separation and nuclear RNA: compartments without boundaries

**Figure 2:**
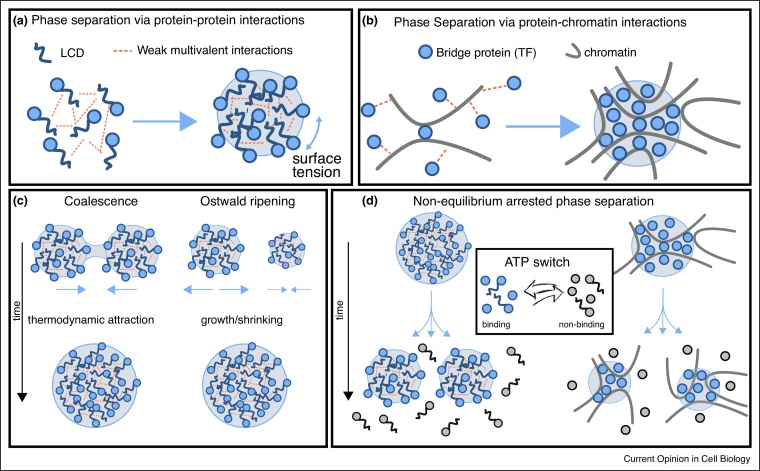
**(a)** Liquid-liquid phase separation via protein-protein interactions yields membraneless bodies which become spherical driven by surface tension. **(b)** Polymer-polymer phase separation via protein-chromatin interactions yields membraneless aggregates of proteins containing chromatin in their interior (TF = transcription factor). **(c)** Thermodynamics-driven droplet coarsening and Ostwald ripeninig [27]) yields slowly or non-recoverable droplets under FRAP. **(d)** Non-equilibrium arrested phase separation via ATP-switch [28^••^] yields non-growing droplets whose constituents are ever-recycling: via FRAP they appear as recoverable bodies with free diffusing and bound sub-populations of proteins.

The eukaryotic nucleus is a complex and heterogeneous environment in which a multitude of biological processes occur simultaneously. One requirement for the viability of a cell is that these processes should not interfere with one another: one way to achieve this is to compartmentalise operations [29]. By staining different proteins, one can readily see a plethora of sub-nuclear structures, including Cajal bodies, nuclear speckles, RNP granules and nucleoli. These structures appear as nuclear compartments without boundaries [30^•^] and some of them require RNA to be formed [19]. One increasingly mentioned mechanism through which these structures can assemble is via phase separation [31, 32, 33, 34, 35, 36]. This is a topic of current debate which has been recently well reviewed (e.g., Refs. [26,37],[30^•^]) and recent evidence suggest that this phenomenon may play important regulatory roles in transcription [38,39],[40^•^]. Here we emphasise the potential different types of phase separation of nuclear proteins. In one case, also called liquid-liquid phase separation [30^•^], clusters of proteins coarsen to form a condensate by weak mutlivalent self-attraction (Figure 2a). This might be the case for phosphorylated HP1 which associates in vitro to form droplets [36]; in the other, proteins that can multi-valently bind to chromatin segments are effectively attracted to one-another through entropic forces, even though they display no self-attraction [41] (Figure 2b). This polymer-polymer phase separation [30^•^], or “bridging-induced attraction” [41], drives a type of demixing which requires a polymer substrate, such as chromatin, in order to occur. This pathway is thus more difficult to prove in vitro as it requires a model of synthetic chromatin. Very recent evidence appear to suggest that both pathways can take place in vivo [42]. Additionally, ubiquitous transcription factors that are known to bind chromatin, such as Polycomb Repressive Complexes or HP1, may be conjectured to give rise to nuclear bodies through bridging-induced attraction in vivo [43].

While some nuclear bodies show liquid-like properties such as coarsening (Figure 2c), their behaviour cannot be fully described by thermodynamic models [29,44] and accounting for the constant influx and consumption of ATP is thus required. The role of non-equilibrium processes on the formation, and phase separation, of membraneless nuclear compartments is only starting to being addressed (see Figure 2d) [28^••^] [45^•^] [46^•^]. Mathematical models show that the consumption of energy via ATP consumption can arrest thermodynamics-driven full phase separation and can stabilise a state in which proteins form micro-phase separated aggregates, i.e. a multitude of non-growing droplets with finite size and made of ever-recycling components (Figure 2d). Such a situation is not achievable in equilibrium systems, where thermodynamic coalescence and Ostwald ripening (Figure 2c), the same controlling the demixing of oil in water, would push the system to minimise the interface between different phases [27,47].

A recent intriguing development in this picture is that RNA plays a non-trivial role in determining the phase separation properties of a multitude of proteins. In particular, low concentration of RNA appears to promote the phase separation of RNA binding proteins or proteins with intrinsically disordered regions such as FUS [48^••^] and hnRNP [49].

As mentioned previously, the hnRNP family of proteins possesses RNA-binding domains and SAF-A also has an AAA+ domain with ATPase activity [15^••^]. This suggests that its phase separation properties are expected to be dependent on both RNA and ATP. While far from being characterised in full, we hypothesise that the phase separation phenomenology of RNA-binding proteins with ATPase domains will be more pervasive than those of other non-ATP-consuming proteins such as HP1. The characterisation of these features, and the understanding of their implication on biological processes, chromatin organisation and the concept of dynamic mesh presented above, remain an exciting challenge for the near future to be tackled via experiments and non-equilibrium mathematical models.

## Micro-phase separated hydro-gels defend transcription micro-environments

**Figure 3:**
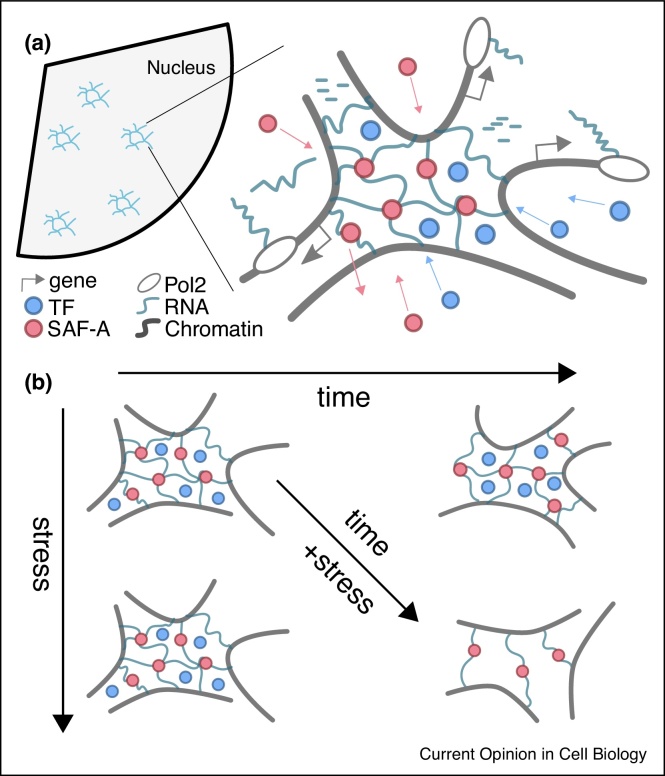
**(a)** The self-assembly and renewal of a dynamic mesh is driven by nuclear RNA (e.g., introns and lncRNA) and SAF-A. In turn, this mesh may facilitate transcription by recruiting or trapping transcription factors (TF). **(b)** The mesh is dynamic, so that it changes in time (fluid-like) but it is resilient to acute mechanical stress (solid-like). Mechanical stress that is prolonged beyond the time-scale of the network renewal affects the structure and hence its function (e.g. can no longer recruit/trap TFs).

The bimodal nature of SAF-A, i.e. displaying both a specific RNA-binding domain and an intrinsically disordered region which can drive phase separation through non-specific interactions [25], is particularly suited to the assembly of a localised structure that must resist strain, such as a phase separated (hydro-)gel. In this model, SAF-A is locally concentrated via phase-separation and it then forms oligomers in presence of RNA; these elongated fibres then cross-link together to form a resilient mesh with high internal water content. At the same time, SAF-A also displays ATPase activity which appears to trigger its de-oligomerisation [15^••^]: this is expected to affect the material properties of a hydro-gel so to make it effectively fluid on time-scales much longer than SAF-A (de-)oligomerisation and effectively solid, or resilient to stress, on shorter time-scales (Figure 3).

While this model remains to be proved both in vivo and in vitro, it is tempting to connect it to other recent findings. Indeed, SAF-A depletion has been shown to mainly affect euchromatin compaction and to leave heterochromatin-rich loci largely unaffected [15^••^], [23^•^]. Because of this, we may speculate that a SAF-A based hydro-gel may be preferentially located at generic active chromatin loci in turn contributing to maintain their decompacted state (Figure 3) [50], [51^••^]. This conformational state can only be maintained by a 3D micro-environment that can sustain external stress originating from the natural tendency of chromatin to self-associate [52, 53, 54]. Whether such micro-environment provides other benefits to transcription remains to be discovered. For instance, it is tempting to speculate that the concept of “sticky caves”, seen through the dynamics of transcription factors such as SOX2 [55], may reflect the presence of an underlying fractal structure such as that of a gel nearby to transcriptionally active chromatin regions (Figure 3). At the same time, we can speculate that the history-dependent recovery of RNA-production after repeated stimuli in optogenetic experiments may also be seen as indicative of the assembly of a micro-environment that promotes transcription after the first stimulus [56^•^]. Additionally, recent evidence suggest that transcription inhibition leads to a reduction in chromatin dynamics [57], which is compatible with the destabilisation of a 3D micro-environment that involves nuclear RNA and constrains chromatin motion.

The final proof of the presented model would be to test the phase separation and material properties of hnRNP proteins and SAF-A in vitro. The “rheology” (from the Greek “panta rhei”, i.e. everything flows) of the self-assembled material, whether liquid, solid or something in between, under different RNA conditions will shed much light into the functional role of this class of proteins. The biophysical characterisation of RNA-dependent phase separation would open a new mechanistic understanding of how hnRNPs, and other RNA-binding proteins, may regulate chromatin structure and genome function through the interaction with nuclear and non-coding RNAs.

In conclusion, we argue that nuclear RNA and associated proteins, such as SAF-A, are key regulators of genome architecture which need to be better understood to achieve a comprehensive picture of nuclear organisation. We believe that the concept of arrested phase separation via non-equilibrium (ATP-driven) mechanisms and interactions with nuclear RNA is a powerful model to describe the formation of ever-recycling membraneless compartments with self-limiting sizes, i.e. nuclear bodies. Furthermore, we speculate that similar mechanisms may underlie the self-assembly of a dynamic nuclear hydro-gel which supports and defends large-scale chromatin structure and transcriptionally-active micro-environments.

The authors declare no conflict of interest.
